# A prediction model of atrial fibrillation recurrence after first catheter ablation by a nomogram: HASBLP score

**DOI:** 10.3389/fcvm.2022.934664

**Published:** 2022-09-06

**Authors:** Wenqiang Han, Yan Liu, Rina Sha, Huiyu Liu, Aihua Liu, Kellina Maduray, Junye Ge, Chuanzhen Ma, Jingquan Zhong

**Affiliations:** ^1^Department of Cardiology, The Key Laboratory of Cardiovascular Remodeling and Function Research, Chinese Ministry of Education, Chinese National Health Commission and Chinese Academy of Medical Sciences, The State and Shandong Province Joint Key Laboratory of Translational Cardiovascular Medicine, Qilu Hospital, Cheeloo College of Medicine, Shandong University, Jinan, China; ^2^Department of Cardiology, Qilu Hospital (Qingdao), Cheeloo College of Medicine, Shandong University, Qingdao, China

**Keywords:** atrial fibrillation, catheter ablation, recurrence, prediction model, nomogram

## Abstract

**Background:**

At present, catheter ablation is an effective method for rhythm control in patients with atrial fibrillation (AF). However, AF recurrence is an inevitable problem after catheter ablation. To identify patients who are prone to relapse, we developed a predictive model that allows clinicians to closely monitor these patients and treat them with different personalized treatment plans.

**Materials and methods:**

A total of 1,065 patients who underwent AF catheter ablation between January 2015 and December 2018 were consecutively included in this study, which examines the results of a 2-year follow-up. Patients with AF were divided into development cohort and validation cohort. Univariate and multivariate analyses were carried out on the potential risk factors. Specific risk factors were used to draw the nomogram according to the above results. Finally, we verified the performance of our model compared with CHADS2 and CHA_2_DS_2_-Vasc scores by receiver operating characteristic (ROC) curve and calibration curve and plotted the decision analysis curve (DAC).

**Results:**

A total of 316 patients experienced AF recurrence. After univariate and multivariate analyses, AF history (H), age (A), snoring (S), body mass index (BMI) (B), anteroposterior diameter of left atrial (LA) (L), and persistent AF (P) were included in our prediction model. Our model showed a better performance compared with CHADS2 and CHA2DS2-Vasc scores, and the area under ROC curve (95%CI) was 0.7668 (0.7298–0.8037) vs. 0.6225 (0.5783–0.6666) and 0.6267 (0.5836–0.6717).

**Conclusion:**

We established a nomogram (HASBLP score) for predicting AF recurrence after the first catheter ablation at a 2-year follow-up, which can be used as a tool to guide future follow-up of patients. However, its usefulness needs further validation.

## Introduction

Atrial fibrillation (AF) is the most common arrhythmia in adults worldwide (1). AF is associated with substantial morbidity and mortality, placing a significant burden on patients, families, and healthcare systems. The estimated prevalence of AF in adults is between 2% and 4% (2), and it will continue to rise due to the lengthening of life expectancy and improvement of screening methods (3–5). Catheter ablation of AF has been recommended by several important guidelines as an effective rhythm control strategy (1, 6), since it reduces hospitalization rate and improves the quality of life; however, its most significant disadvantage is recurrence. Recurrence of AF would not only affect enthusiasm for catheter ablation in patients with AF but also bring some potential risks.

According to several studies, both individuals with and without an AF recurrence have a different chance of developing thromboembolism (7–10). Nevertheless, AF recurrence is usually asymptomatic (11), causing an unawareness of the episode in a considerable number of patients. Therefore, the continued use of oral anticoagulation in patients with AF after catheter ablation remains controversial (12). The objective world needs a prediction model to predict the probability of AF recurrence to guide the follow-up after AF catheter ablation. At the same time, a recurrence prediction model could also assist in screening patients undergoing catheter ablation. Several predictors of arrhythmia recurrence, including left atrial (LA) size, LA fibrosis, non-paroxysmal AF, hypertension, and sleep apnea syndrome, had been proposed in previous studies (13). CHADS2 and CHA2DS2-Vasc scores have been shown to predict the recurrence of AF to some extent (14); however, as a prediction model, the result does not seem ideal.

In this study, we attempted to develop a predictive model for recurrence after the first catheter ablation in patients with AF by following and reviewing clinical data from those with AF and compared our predictive model with the CHADS2 and CHA_2_DS_2_-Vasc score models.

## Materials and methods

### Study design

We aimed to establish a prediction model with the outcome of 2-year follow-up of patients with AF after catheter ablation. This study was based on data from a prospective observational study (Chinese Clinical Trial Registry: ChiCTR-OCH-14004674) of patients who underwent ablation at our center. The primary endpoint of this study was AF recurrence, defined as symptomatic or documented AF, atrial flutter, or atrial tachycardia >30s after a 3-month blanking period after the first catheter ablation.

### Patients selection

All patients who underwent AF catheter ablation between January 2015 and December 2018 were consecutively included in this study unless they met any of the following exclusion criteria: (1) patients with a previous history of catheter ablation; (2) patients with < 24 months of follow-up; or (3) patients who underwent cardiac surgery during the follow-up period. Prior to catheter ablation, coronary computed tomography (CTA) or transesophageal echocardiography was performed to rule out cardiac thrombosis.

### Data collection

Age, gender, course of AF, type of AF, history of related diseases, LA size, and left ventricular ejection fraction (LVEF) were collected before the procedure, and AF history (years) was found based on medical records or according to patient-reported time of first documented AF. The types of AF were divided into paroxysmal AF and persistent AF (e.g., long-standing persistent AF). LA size was represented by its anteroposterior diameter measured by echocardiography. Patients with heart failure were defined as ≥ class 2 (classification of NYHA heart function) according to the admission diagnosis.

Oral anticoagulant (OAC; warfarin, rivaroxaban, or dabigatran) was used at least 3 months after catheter ablation. All patients had a follow-up of at least 24 months after the procedure. Documented AF was evaluated by electrocardiography (ECG) and a 24-h Holter monitoring at the first, third, and sixth months and every 6 months thereafter. If the patient did not show up for a scheduled follow-up, our office contacted them telephonically to recommend 24-h Holter monitoring at the local hospital and collect information on recurrence. Time and outcome of primary events were recorded during follow-up.

### Statistical analysis and nomogram

Data analysis was performed using IBM SPSS Statistic 25 and R,^1^ and the significance level was set at *p* < 0.05. Two-thirds of all patients were taken as development cohort and one-third of patients as validation cohort by random sampling. The rank sum test was used for numerical variables with non-normal distribution, independent *t*-test random was used for numerical variables with normal distribution, and categorical variables were tested by chi-square test (χ^2^ test). Univariate analysis was performed using the abovementioned methods and variables with *p* < 0.05 were included in the subsequent logistic regression. Iteratively reweighted least squares (IWLS) were used to fit the logistic regression model based on development cohort data (model 1); then, according to the results of logistic regression, the variables with *p* < 0.05 were selected to form model 2.

Nomogram was constructed in accordance with the results of model 2. A nomogram is valuable because it converts anticipated probabilities into points on a scale of 0–100 in a user-friendly graphic interface (15). The total number of points accumulated by various factors corresponds to a patient’s expected likelihood (16, 17). The point system ranks effect estimates irrespective of statistical significance, and it is modified by the existence of other factors.

The total score of the nomogram was the sum of the corresponding score assigned to each risk factor, which corresponds to the recurrence risk.

### Prediction performance of the nomogram

Receiver operating characteristic (ROC) and calibration curves were plotted using development cohort data and validation cohort data, respectively. Subsequently, a decision curve analysis (DCA) diagram was drawn from development cohort data to guide clinical decision-making. Risk factors included in CHADS_2_ and CHA_2_DS_2_-Vasc scores were used to form model CHADS_2_ and CHA_2_DS_2_-Vasc. Using development cohort data, ROC curves for the CHADS2 and CHA2DS2-Vasc models were created, and the area under the curve (AUC) was calculated.

## Results

### Basic information

As shown in Table 1, a total of 1,065 patients (no recurrence: recurrence = 749:316) were included in this study; the development cohort consisted of 710 patients (no recurrence: recurrence = 490:220), while the validation cohort consisted of 355 patients (no recurrence: recurrence = 259:96). Non-normally distributed continuous data were presented as median (Q1, Q3), normally distributed data were presented as mean ± standard (SD), and categorical variables were presented as percentages. Finally, after univariate analysis, age (*p* < 0.01), body mass index (BMI; *p* < 0.01), AF history (*p* < 0.01), snoring (*p* < 0.01), hypertension (*p* = 0.04), coronary heart disease (*p* < 0.01), diabetes (*p* = 0.01), heart failure (*p* < 0.01), valve diseases (*p* = 0.02), cardiomyopathy (*p* = 0.03), persistent AF (*p* < 0.01), and the anteroposterior diameter of the LA (*p* < 0.01) were found to be statistically significant with AF recurrence.

**TABLE 1:** Baseline characteristics of all patients.

	Total cohort			Development cohort				Validation cohort		
Variables	Total (*n* = 1,065)	No recurrence (*n* = 749)	Recurrence (*n* = 316)	Total (*n* = 710)	No recurrence (*n* = 490)	Recurrence (*n* = 220)	*p*	Total (*n* = 355)	No recurrence (*n* = 259)	Recurrence (*n* = 96)
Sex, male (%)	674 (63)	480 (64)	194 (61)	443 (62)	311 (63)	132 (60)	0.42	231 (65)	169 (65)	62 (65)
Age, Median (Q1, Q3)	61 (53, 67)	61 (53, 66)	63 (55, 69)	61 (53, 68)	60 (53, 66.75)	63.5 (57, 70)	< 0.01	61 (53, 66)	61 (53, 66)	61 (54, 66.25)
Snoring, n (%)	475 (45)	271 (36)	204 (65)	310 (44)	168 (34)	142 (65)	< 0.01	165 (46)	103 (40)	62 (65)
BMI, Median, (Q1, Q3)	26.34 (24.34, 28.65)	25.88 (24, 27.92)	27.66 (25.34, 29.97)	26.24 (24.31, 28.54)	25.79 (23.91, 27.73)	27.54 (25.49, 29.61)	< 0.01	26.45 (24.46, 29.00)	26.17 (24.16, 28.13)	27.99 (24.89, 30.38)
AF history, Median (Q1, Q3)	2 (0.4, 5)	1.5 (0.3, 4)	3 (1, 6.1)	2 (0.4, 5)	1.3 (0.3, 4)	3 (1, 7)	< 0.01	2 (0.5, 5)	2 (0.4, 4)	3 (0.8, 5.3)
Hypertension, n (%)	479 (45)	320 (43)	159 (50)	319 (45)	207 (42)	112 (51)	0.04	160 (45)	113 (44)	47 (49)
CHD, n (%)	211 (20)	132 (18)	79 (25)	154 (22)	91 (19)	63 (29)	< 0.01	57 (16)	41 (16)	16 (17)
Diabetes, n (%)	144 (14)	89 (12)	55 (17)	96 (14)	55 (11)	41 (19)	0.01	48 (14)	34 (13)	14 (15)
Heart failure, n (%)	70 (7)	32 (4)	38 (12)	55 (8)	24 (5)	31 (14)	< 0.01	15 (4)	8 (3)	7 (7)
Cardiomyopathy, n (%)	23 (2)	12 (2)	11 (3)	13 (2)	5 (1)	8 (4)	0.03	10 (3)	7 (3)	3 (3)
Valvular heart disease, n (%)	27 (3)	12 (2)	15 (5)	19 (3)	8 (2)	11 (5)	0.02	8 (2)	4 (2)	4 (4)
TIA/stroke, n (%)	76 (7)	55 (7)	21 (7)	52 (7)	35 (7)	17 (8)	0.90	24 (7)	20 (8)	4 (4)
Renal disease, n (%)	14 (1)	9 (1)	5 (2)	10 (1)	6 (1)	4 (2)	0.54	4 (1)	3 (1)	1 (1)
Vascular disease, n (%)	94 (9)	62 (8)	32 (10)	63 (9)	41 (8)	22 (10)	0.57	31 (9)	21 (8)	10 (10)
Smoke, n (%)	331 (31)	244 (33)	87 (28)	214 (30)	158 (32)	56 (25)	0.08	117 (33)	86 (33)	31 (32)
Drink, n (%)	272 (26)	201 (27)	71 (22)	177 (25)	131 (27)	46 (21)	0.12	95 (27)	70 (27)	25 (26)
persistent AF, n (%)	409 (38)	250 (33)	159 (50)	270 (38)	166 (34)	104 (47)	< 0.01	139 (39)	84 (32)	55 (57)
LA, Median (Q1, Q3)	40 (37, 43)	39 (36, 43)	42 (39, 45)	40 (37, 43)	39 (36, 42.75)	42 (39, 45)	< 0.01	40 (36, 43)	39 (36, 43)	41.5 (38, 45)
LVEF, Median (Q1, Q3)	0.6 (0.6, 0.63)	0.6 (0.6, 0.64)	0.6 (0.59, 0.63)	0.6 (0.6, 0.64)	0.6 (0.6, 0.64)	0.6 (0.59, 0.63)	0.50	0.6 (0.59, 0.63)	0.6 (0.6, 0.63)	0.6 (0.58, 0.6)
CHA_2_DS_2_-Vasc score*, mean ± SD	1.67 ± 1.35	1.57 ± 1.31	1.91 ± 1.41	1.71 ± 1.38	1.56 ± 1.33	2.05 ± 1.45	–	1.60 ± 1.28	1.60 ± 1.29	1.60 ± 1.28

SD, standard deviation; BMI, body mass index; CHD, coronary heart disease; TIA, transient ischemic attack; LA, left atrium; LVEF, left ventricular ejection fraction; Q1, Q3: 25% and 75% quartile.

*CHA_2_DS_2_-Vasc score was the result of multiple risk factors, it was not included in the univariate and multivariate analysis.

### Atrial fibrillation recurrence nomogram and other prediction models

The abovementioned statistically significant risk factors were used to build model 1 (recurrence∼age + snoring + BMI + AF history + hypertension + coronary heart disease + diabetes + heart failure + valve diseases + cardiomyopathy + persistent AF + LA).

**FIGURE 1:**
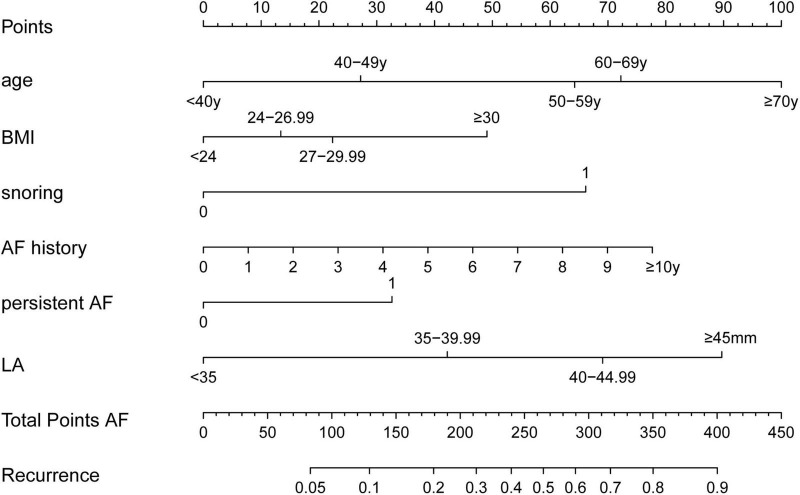
Atrial fibrillation (AF) recurrence nomogram. The nomogram was developed in the development cohort. The total score of the nomogram was the sum of the corresponding score assigned to each risk factor, and the total score corresponds to the recurrence risk.

According to the results of logistic regression, the variables with *p* < 0.05 were selected to form model 2 (recurrence∼age + snoring + BMI + AF history + persistent AF + LA) to facilitate the clinical application, age was divided into five segments (< 40 years, 40–49 years, 50–59 years, 60–69 years, and ≥ 70 years), LA anteroposterior diameter was divided into four segments (<35 mm, 35–39.99 mm, 40–44.99 mm, and ≥ 45 mm), and BMI was divided into four segments according to the Chinese standard (< 24, 24–26.99, 27–29.99, and ≥ 30). In addition, the result of model 2, which we termed the HASBLP score (AF history, age, snoring, BMI, LA, and persistent AF), was used to plot the nomogram (Figure 1).

### Prediction performance of the nomogram

**FIGURE 2:**
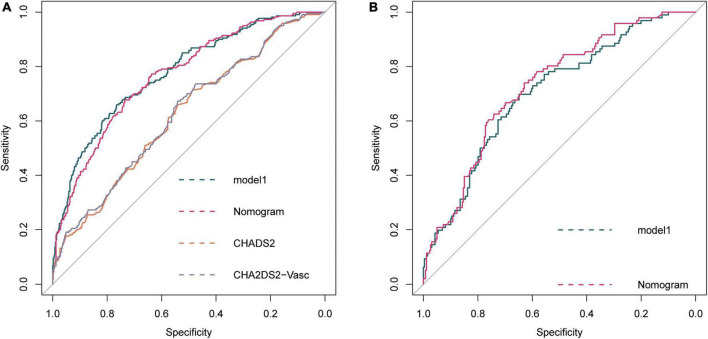
Receiver operating characteristic (ROC) curve of prediction model. **(A)** Development cohort; **(B)** Validation cohort. Model 1: Recurrence∼ age + snoring + BMI + AF history + hypertension + coronary heart disease + diabetes + heart failure + valve diseases + cardiomyopathy + persistent AF + LA. HASBLP: Recurrence∼age + snoring + BMI + AF history + persistent AF + LA. CHADS2: Recurrence∼heart failure + hypertension + age + diabetes + stroke. CHA2DS2-Vasc: Recurrence∼heart failure + hypertension + age + diabetes + stroke + vascular disease + female.

**FIGURE 3:**
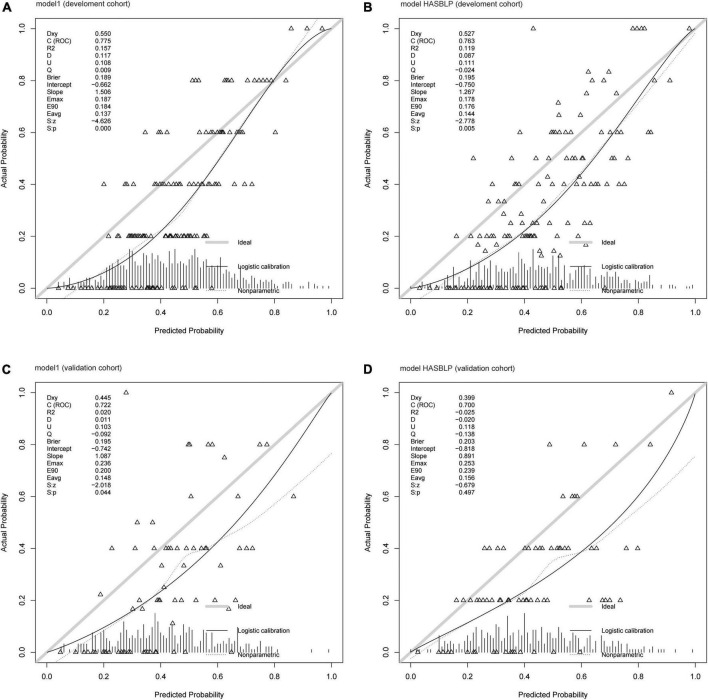
Calibration curve of prediction models. **(A)** Model 1 (development cohort); **(B)** model HASBLP (development cohort); **(C)** model 1 (validation cohort); and **(D)** model HASBLP (validation cohort).

**FIGURE 4:**
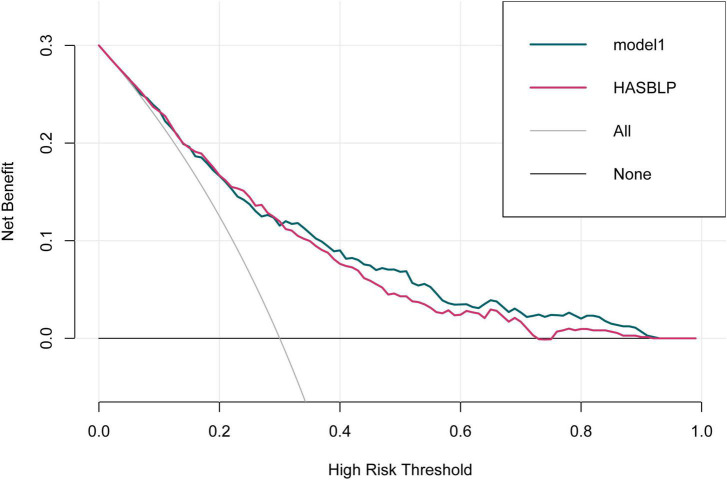
Decision curve analysis curve of model HASBLP.

The ROC curve of model 1, model HASBLP, and model CHADS_2_ and CHA_2_DS_2_-Vasc in the development cohort data is shown in Figure 2A, and their AUCs, shown in Table 2, were 0.7766 (95%CI, 0.7397–0.8135), 0.7668 (95%CI, 0.7298–0.8037), 0.6225 (95%CI, 0.5783–0.6666), and 0.6267 (95%CI, 0.5836–0.6717), respectively. Based on this result, we found that the CHADS_2_ and CHA_2_DS_2_-Vasc scores predict AF recurrence with suboptimal results, and the HASBLP score was better able to predict AF recurrence. The ROC curves of model 1 and HASBLP score with validation cohort data are shown in Figure 2B; calibration curves of model 1 and HASBLP score with development and validation cohort data are shown in Figures 3A–D. The analysis of DCA showed that the recurrence probability of patients was in the range of about 5 to 80%, and this model has the highest accuracy and net benefit in clinical application (Figure 4).

**TABLE 2:** Area under curve of receiver operating curve.

		AUC (95%CI)	Specificity (%)	Sensitivity (%)	Accuracy (%)
Development cohort	Model 1	0.7766 (0.7397–0.8135)	76.53	65.91	73.24
	HASBLP	0.7668 (0.7298–0.8037)	73.47	67.73	71.69
	CHADS_2_	0.6225 (0.5783–0.6666)	54.29	65.91	57.89
	CHA_2_DS_2_-Vasc	0.6267 (0.5836–0.6717)	54.08	67.27	58.17
Validation cohort	Model 1	0.7038 (0.6441–0.7634)	64.84	69.79	66.20
	HASBLP	0.7264 (0.6697–0.7830)	62.93	73.96	65.92

## Discussion

Using clinical data and follow-up results of patients with AF in our center, we constructed a prediction model to predict AF recurrence after the first catheter ablation, which showed better performance compared with CHADS_2_ and CHA_2_DS_2_-Vasc scores. Several other scores, such as DR-FLASH (AUC 0.801) (18), CAAP-AF (AUC 0.691) (19), ATLAS (AUC 0.750) (20), APPLE (AUC 0.634) (21), and MB-LATER (AUC 0.782) (22), have shown good predictive effectiveness in their respective studies. However, there are differences in the overall variables included in our study compared with these studies, so it is difficult to compare them directly.

It is reported that age is the most common risk factor for AF recurrence in several trials and other prediction models (20, 21, 23, 24), which indicated that younger patients with AF may have a lower risk of recurrence following catheter ablation and thereby may be more suitable for the procedure. Being overweight or obese not only promotes the development of AF but also increases the risk of recurrence after catheter ablation (25–28). This may be associated with an increase in epicardial adipose tissue, which is a source of pro-inflammatory adipocytokines, leading to microvascular dysfunction and myocardial fibrosis (29). Inflammation has been proven to affect the occurrence of AF through multiple pathways (30). Obesity was also accompanied by other cardiovascular disease risk factors, such as hypertension, diabetes, and sleep apnea syndrome (31, 32), so weight loss could not only reduce the AF load (33) but also reduce AF recurrence after catheter ablation (34). Other studies have mentioned a history of AF as a risk factor for recurrence of AF after catheter ablation (35, 36); this may be due to the changes in the atrial matrix caused by risk factors over time. Jens Cosedis Nielsen’s trial proved that the efficacy of catheter ablation in patients with paroxysmal AF is better than that of patients with persistent AF (37). Although catheter ablation is effective for patients with persistent AF, the risk of recurrence is higher than that of patients with paroxysmal AF. Age, AF burden, obesity, smoking, renal insufficiency, and other cardiovascular risk factors promote atrial remodeling (38). While atrial enlargement is more likely a result of multiple factors, it often reflects atrial fibrosis. A study on MRI evaluation of atrial fibrosis and AF recurrence suggested that atrial fibrosis may be an independent risk factor for recurrence of AF after catheter ablation (39). In our study, the LA anteroposterior diameter measured by echocardiography represented the atrial size, which was slightly less accurate than the LA volume measured by CT or echocardiography. Still, it could increase the applicability of the model. Previous studies have shown that snoring is related to sleep apnea (SA) (40, 41). While snoring does not represent SA, habitual snoring is often a form of SA (42). Obstructive sleep apnea syndrome (OSAS) could promote the occurrence and progress of cardiovascular diseases, such as hypertension and arrhythmia (40, 41). A meta-analysis had shown OSAS could promote AF recurrence (43), and continuous positive airway pressure ventilation had a positive effect on preventing AF recurrence, which may be related to the correction of hypoxemia during sleep (44). In addition, a recent study showed that a healthy sleep pattern is associated with lower risks of AF and bradyarrhythmia, independent of traditional risk factors (45). An AF patient with snoring may be comorbid with OSAS or hypoxemia (44); however, some patients rarely get a proper diagnosis and treatment for a variety of reasons. Therefore, in our prediction model, OSAS was replaced by snoring. Snoring during sleep may be inaccurate and ambiguous compared with OSAS, but snoring as a common phenomenon is more practical in our opinion.

The present model might guide patients with AF to correct reversible risk factors after catheter ablation, such as weight loss, improvement of hypoxemia during sleep, and drug intervention for the process of cardiac fibrosis. It proposes that early treatment with catheter ablation not only allows for better symptom control but may also reduce the probability of AF recurrence. With the exploration of recurrence risk factors and the prediction models, we could screen patients who intend to undergo catheter ablation. For patients with a high risk of recurrence, catheter ablation should be carefully examined.

In addition, a long-term use of OAC or cessation of OAC after 3 months post-ablation remains controversial (46, 47). In our previous study, we concluded that cessation of OAC in non-recurrent AF may be reasonable; however, cessation appeared unsafe in recurrent AF with a high thromboembolism risk (10). With the help of the prediction model, patients at a high risk of recurrence could be identified after catheter ablation, allowing us to monitor these patients closely and encourage them to continue OAC.

There are several other limitations to our study. Due to following up with 24-h Holter ECG only, it might miss some patients with asymptomatic recurrence, which was inevitable in our study. This was a single-center study and the sample size should be expanded for more robust conclusions. Our prediction model needs to be verified by multicenter research. In this model, two variables (LA size and snoring) may be questioned for inaccuracies.

## Conclusion

This study established a model (HASBLP score) for predicting AF recurrence after the first catheter ablation, which can be used as a tool to guide patients’ follow-up. Compared with CHADS_2_ and CHA_2_DS_2_-Vasc scores, this model showed a better performance in predicting AF recurrence. However, its role requires further validation.

## Data availability statement

The raw data supporting the conclusions of this article will be made available by the authors, without undue reservation.

## Ethics statement

The studies involving human participants were reviewed and approved by Medical Ethics Committee of Qilu Hospital of Shandong University. Written informed consent for participation was not required for this study in accordance with the national legislation and the institutional requirements.

## Author contributions

WH and YL: conceptualization, methodology, writing—original draft, writing—review and editing, investigation, and visualization. RS, HL, AL, KM, JG, and CM: investigation and resources. JZ: conceptualization, writing—review and editing, supervision, and funding acquisition. All authors contributed to the article and approved the submitted version.

## Footnotes

1
https://www.r-project.org
